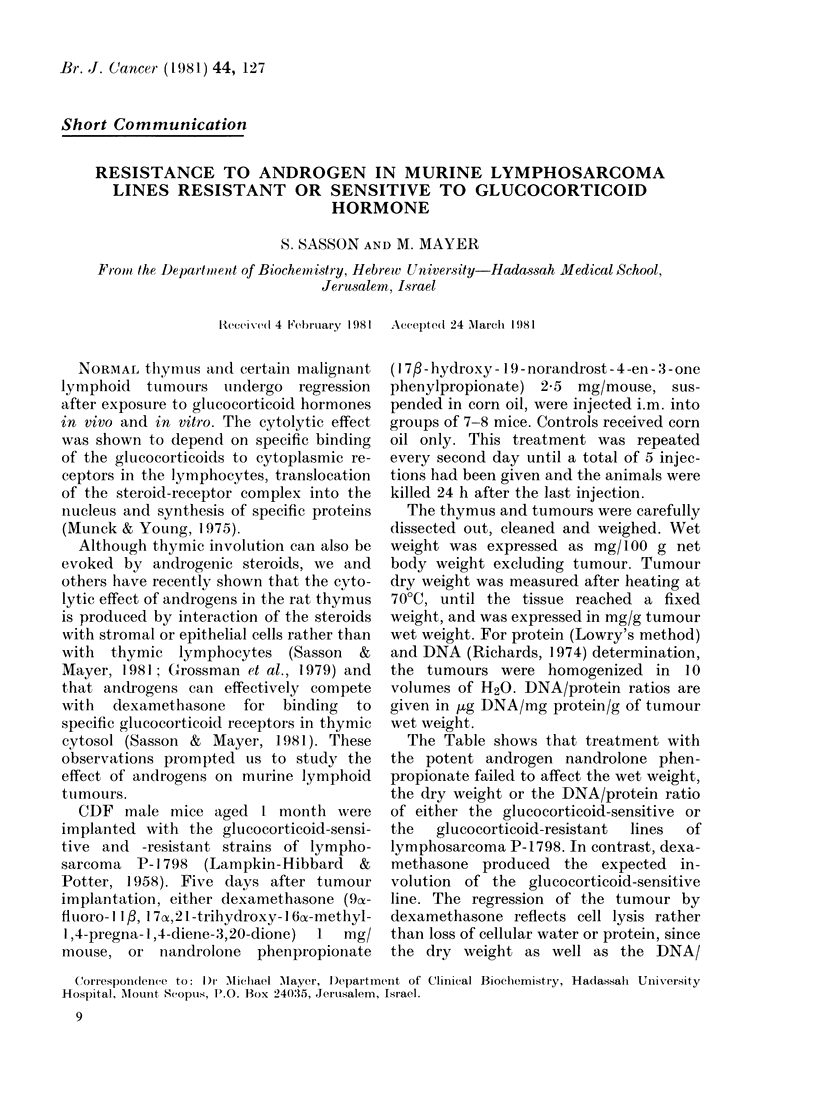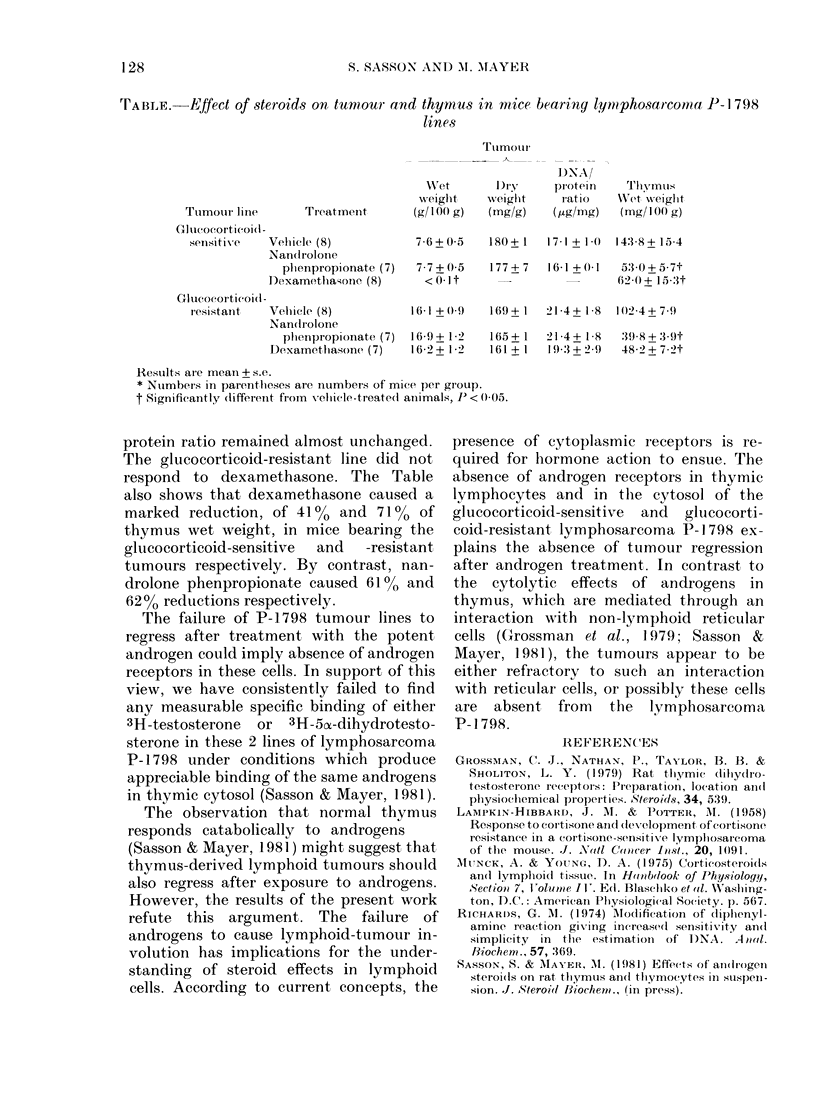# Resistance to androgen in murine lymphosarcoma lines resistant or sensitive to glucocorticoid hormone.

**DOI:** 10.1038/bjc.1981.158

**Published:** 1981-07

**Authors:** S. Sasson, M. Mayer


*Br. J. Cancer* (1981) **44**, 127

### *Short Communication*

### RESISTANCE TO ANDROGEN IN MURINE LYMPHOSARCOMA LINES RESISTANT OR SENSITIVE TO GLUCOCORTICOID HORMONE

S. SASSON AND M. MAYER

*From the Department of Biochemistry, Hebrew University—Hadassah Medical School, Jerusalem, Israel*

Received 4 February 1981 Accepted 24 March 1981

NORMAL thymus and certain malignant lymphoid tumours undergo regression after exposure to glucocorticoid hormones *in vivo* and *in vitro*. The cytolytic effect was shown to depend on specific binding of the glucocorticoids to cytoplasmic receptors in the lymphocytes, translocation of the steroid-receptor complex into the nucleus and synthesis of specific proteins (Munck & Young, 1975).

Although thymic involution can also be evoked by androgenic steroids, we and others have recently shown that the cytolytic effect of androgens in the rat thymus is produced by interaction of the steroids with stromal or epithelial cells rather than with thymic lymphocytes (Sasson & Mayer, 1981; Grossman *et al.*, 1979) and that androgens can effectively compete with dexamethasone for binding to specific glucocorticoid receptors in thymic cytosol (Sasson & Mayer, 1981). These observations prompted us to study the effect of androgens on murine lymphoid tumours.

CDF male mice aged 1 month were implanted with the glucocorticoid-sensitive and -resistant strains of lymphosarcoma P-1798 (Lampkin-Hibbard & Potter, 1958). Five days after tumour implantation, either dexamethasone (9α-fluoro-11β, 17α,21-trihydroxy-16α-methyl-1,4-pregna-1,4-diene-3,20-dione) 1 mg/mouse, or nandrolone phenpropionate (17β-hydroxy-19-norandrost-4-en-3-one phenylpropionate) 2·5 mg/mouse, suspended in corn oil, were injected i.m. into groups of 7–8 mice. Controls received corn oil only. This treatment was repeated every second day until a total of 5 injections had been given and the animals were killed 24 h after the last injection.

The thymus and tumours were carefully dissected out, cleaned and weighed. Wet weight was expressed as mg/100 g net body weight excluding tumour. Tumour dry weight was measured after heating at 70°C, until the tissue reached a fixed weight, and was expressed in mg/g tumour wet weight. For protein (Lowry's method) and DNA (Richards, 1974) determination, the tumours were homogenized in 10 volumes of $H_2O$. DNA/protein ratios are given in μg DNA/mg protein/g of tumour wet weight.

The Table shows that treatment with the potent androgen nandrolone phenpropionate failed to affect the wet weight, the dry weight or the DNA/protein ratio of either the glucocorticoid-sensitive or the glucocorticoid-resistant lines of lymphosarcoma P-1798. In contrast, dexamethasone produced the expected involution of the glucocorticoid-sensitive line. The regression of the tumour by dexamethasone reflects cell lysis rather than loss of cellular water or protein, since the dry weight as well as the DNA/

Correspondence to: Dr Michael Mayer, Department of Clinical Biochemistry, Hadassah University Hospital, Mount Scopus, P.O. Box 24035, Jerusalem, Israel.

TABLE.—*Effect of steroids on tumour and thymus in mice bearing lymphosarcoma P-1798 lines*

| Tumour line | Treatment | Tumour: Wet weight (g/100 g) | Tumour: Dry weight (mg/g) | Tumour: DNA/protein ratio (μg/mg) | Thymus Wet weight (mg/100 g) |
|---|---|---|---|---|---|
| Glucocorticoid-sensitive | Vehicle (8) | 7·6 ± 0·5 | 180 ± 1 | 17·1 ± 1·0 | 143·8 ± 15·4 |
| | Nandrolone phenpropionate (7) | 7·7 ± 0·5 | 177 ± 7 | 16·1 ± 0·1 | 53·0 ± 5·7† |
| | Dexamethasone (8) | < 0·1† | — | — | 62·0 ± 15·3† |
| Glucocorticoid-resistant | Vehicle (8) | 16·1 ± 0·9 | 169 ± 1 | 21·4 ± 1·8 | 102·4 ± 7·9 |
| | Nandrolone phenpropionate (7) | 16·9 ± 1·2 | 165 ± 1 | 21·4 ± 1·8 | 39·8 ± 3·9† |
| | Dexamethasone (7) | 16·2 ± 1·2 | 161 ± 1 | 19·3 ± 2·9 | 48·2 ± 7·2† |

Results are mean ± s.e.
* Numbers in parentheses are numbers of mice per group.
† Significantly different from vehicle-treated animals, $P < 0·05$.

protein ratio remained almost unchanged. The glucocorticoid-resistant line did not respond to dexamethasone. The Table also shows that dexamethasone caused a marked reduction, of 41% and 71% of thymus wet weight, in mice bearing the glucocorticoid-sensitive and -resistant tumours respectively. By contrast, nandrolone phenpropionate caused 61% and 62% reductions respectively.

The failure of P-1798 tumour lines to regress after treatment with the potent androgen could imply absence of androgen receptors in these cells. In support of this view, we have consistently failed to find any measurable specific binding of either $^3$H-testosterone or $^3$H-5α-dihydrotestosterone in these 2 lines of lymphosarcoma P-1798 under conditions which produce appreciable binding of the same androgens in thymic cytosol (Sasson & Mayer, 1981).

The observation that normal thymus responds catabolically to androgens (Sasson & Mayer, 1981) might suggest that thymus-derived lymphoid tumours should also regress after exposure to androgens. However, the results of the present work refute this argument. The failure of androgens to cause lymphoid-tumour involution has implications for the understanding of steroid effects in lymphoid cells. According to current concepts, the presence of cytoplasmic receptors is required for hormone action to ensue. The absence of androgen receptors in thymic lymphocytes and in the cytosol of the glucocorticoid-sensitive and glucocorticoid-resistant lymphosarcoma P-1798 explains the absence of tumour regression after androgen treatment. In contrast to the cytolytic effects of androgens in thymus, which are mediated through an interaction with non-lymphoid reticular cells (Grossman *et al.*, 1979; Sasson & Mayer, 1981), the tumours appear to be either refractory to such an interaction with reticular cells, or possibly these cells are absent from the lymphosarcoma P-1798.

### REFERENCES

GROSSMAN, C. J., NATHAN, P., TAYLOR, B. B. & SHOLITON, L. Y. (1979) Rat thymic dihydrotestosterone receptors: Preparation, location and physiochemical properties. *Steroids*, **34**, 539.

LAMPKIN-HIBBARD, J. M. & POTTER, M. (1958) Response to cortisone and development of cortisone resistance in a cortisone-sensitive lymphosarcoma of the mouse. *J. Natl Cancer Inst.*, **20**, 1091.

MUNCK, A. & YOUNG, D. A. (1975) Corticosteroids and lymphoid tissue. In *Hanbdook of Physiology, Section 7, Volume IV*. Ed. Blaschko *et al.* Washington, D.C.: American Physiological Society. p. 567.

RICHARDS, G. M. (1974) Modification of diphenylamine reaction giving increased sensitivity and simplicity in the estimation of DNA. *Anal. Biochem.*, **57**, 369.

SASSON, S. & MAYER, M. (1981) Effects of androgen steroids on rat thymus and thymocytes in suspension. *J. Steroid Biochem.*, (in press).